# Genomic and gene regulatory signatures of cryptozoic adaptation: Loss of blue sensitive photoreceptors through expansion of long wavelength-opsin expression in the red flour beetle *Tribolium castaneum*

**DOI:** 10.1186/1742-9994-4-24

**Published:** 2007-12-21

**Authors:** Magdalena Jackowska, Riyue Bao, Zhenyi Liu, Elizabeth C McDonald, Tiffany A Cook, Markus Friedrich

**Affiliations:** 1 Department of Biological Sciences, Wayne State University, Detroit, MI 48202 USA; 2 Department of Molecular Biology and Pharmacology, Washington University in St Louis School of Medicine, 3600 Cancer Research Building, St. Louis, MO 63110 USA; 3 Division of Developmental Biology and Department of Pediatric Ophthalmology, Cincinnati Children's Hospital Medical Center, Cincinnati OH 45229 USA; 4 Department of Anatomy and Cell Biology, Wayne State University School of Medicine, Detroit, MI 48201 USA

## Abstract

**Background:**

Recent genome sequence analysis in the red flour beetle *Tribolium castaneum *indicated that this highly crepuscular animal encodes only two single opsin paralogs: a UV-opsin and a long wavelength (LW)-opsin; however, these animals do not encode a blue (B)-opsin as most other insects. Here, we studied the spatial regulation of the *Tribolium *single LW- and UV-opsin gene paralogs in comparison to that of the five opsin paralogs in the retina of *Drosophila melanogaster*.

**Results:**

*In situ *hybridization analysis reveals that the *Tribolium *retina, in contrast with other insect retinas, constitutes a homogenous field of ommatidia that have seven LW-opsin expressing photoreceptors and one UV-/LW-opsin co-expressing photoreceptor per eye unit. This pattern is consistent with the loss of photoreceptors sensitive to blue wavelengths. It also identifies *Tribolium *as the first example of a species in insects that co-expresses two different opsins across the entire retina in violation of the widely observed "one receptor rule" of sensory cells.

**Conclusion:**

Broader studies of opsin evolution in darkling beetles and other coleopteran groups have the potential to pinpoint the permissive and adaptive forces that played a role in the evolution of vision in *Tribolium castaneum*.

## Introduction

**Figure 1:**
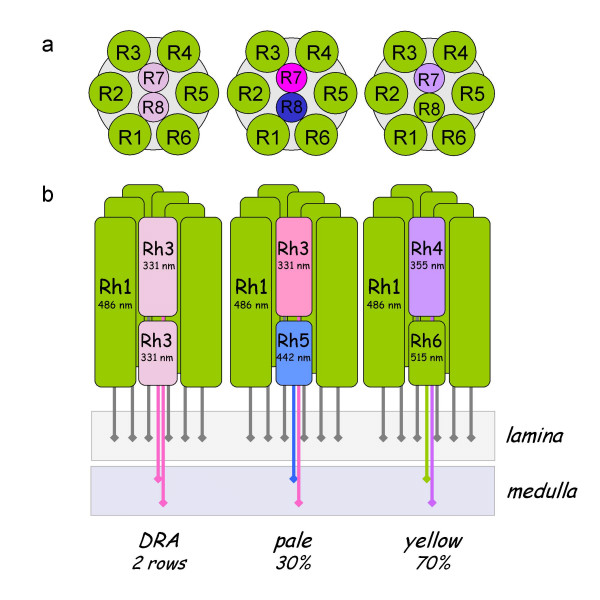
**Differential opsin expression in *Drosophila***. Schematic drawings of photoreceptor cell arrangements in the three types of ommatidia that exist in the retina of *Drosophila melanogaster*, and differ in the choice of differentially expressed opsin paralogs. (a) Cross-section perspective. It should be noted that the R7 and R8 rhabdomeres are represented in the same plane for ease in representation. (b) Sagital section perspective. In pale-type ommatidia, the peripheral photoreceptors R1–R6 express the LW-opsin Rh1, the R7 photoreceptor cell expresses the UV-opsin paralog Rh3, and the R8 photoreceptor cell expresses the B-opsin paralog Rh5. Yellow differ from pale ommatidia by expression of the UV-opsin paralog Rh4 in the R7 cells, and LW-opsin paralog Rh6 in R8. Dorsal rim area (DRA) ommatidia express Rh3 in both the R7 and R8 cell [12].

Photon absorption by visual pigments is the primary event in the conversion of light to a neuronal signal in animal photoreceptor cells [1]. Visual pigments or rhodopsins form by an association of retinal-based chromophores with a special class of G-protein-coupled seven transmembrane receptor proteins (GPCRs). These GPCRs are encoded by a diverse group of opsin gene family paralogs. Most insects encode members of three different opsin subfamilies. Ultraviolet (UV) and blue (B) opsins correlate with maximal photoreceptor sensitivities in the 300–400 and 400–500 nm ranges respectively. Long wavelength (LW) opsin-expressing photoreceptors show sensitivities that range between 480 and 600 nm [2-4]. The differential expression of these diverged opsin paralogs in the photoreceptors of individual eye units (ommatidia) within the compound eye is a structural prerequisite for color discrimination by insects. Moreover, differential opsin expression can correlate with cellular specialization in color and brightness detection in insects. In *Drosophila*, brightness is measured from the input of six peripheral LW-opsin expressing photoreceptors (R1–R6), which project into the first optic neuropil, the lamina. Color vision is facilitated by the central photoreceptors R7 and R8 that express one of the UV-, B-, or LW-opsins and have long axonal projections that synapse into the second optic neuropil, the medulla (Fig. 1) (for review see [4-6]).

The existence of UV-, B- and LW-sensitive photoreceptors in primitive insects implies that trichromatic vision is ancient and highly conserved [2]. Consistent with this, recent genome sequencing projects identified UV-, B- and LW-opsin gene family paralogs in diverse endopterygote insects including silkmoth, mosquito and honeybee [7]. Remarkably, only two opsin gene orthologs representing members of the LW- and UV-opsin groups were detected in the genome of the red flour beetle *Tribolium castaneum*, suggesting the loss of B-opsin during the evolution of this species [7,8] (Bao, Friedrich, in preparation). Like darkling beetles (Tenebrionidae) in general, the stored grain pest *Tribolium castaneum *is highly secretive and avoids broad daylight: all postembryonic life cycle stages hide in tunnels driven through the food substrate [9,10]. The dramatic increase of chemoreceptor genes, compared to diurnal species like honeybee and *Drosophila*, suggests a strong adaptive effect of *Tribolium*'s cryptozoic ecology on its genome and neurobiology [8]. Thus, it is conceivable that a relaxation of color discrimination requirements allowed for the loss of B-opsin in *Tribolium*. However, there are also examples of trichromatic dark active insect species like the tobacco hornworm moth *Manduca sexta *[11].

The highly regulated spatial expression of opsin paralogs in *Drosophila *(Fig. 1) and other endopterygote species allows specific predictions regarding the expression of the remaining two opsin paralogs in *Tribolium *if B-opsin is missing [5,12]. That is, in the absence of B-opsin, the ancestral B-opsin expression niche in a fraction of the central R7 and R8 cells should be replenished by either UV- or LW-expression. By examining the expression patterns of the identified LW- and UV-opsin genes in the *Tribolium *retina, we sought to answer if the spatial regulation of these genes was consistent with a loss of B-opsin, and if so, which specific changes in the regulation of either LW- or UV-opsin expression would be associated with it.

## Results

**Figure 2:**
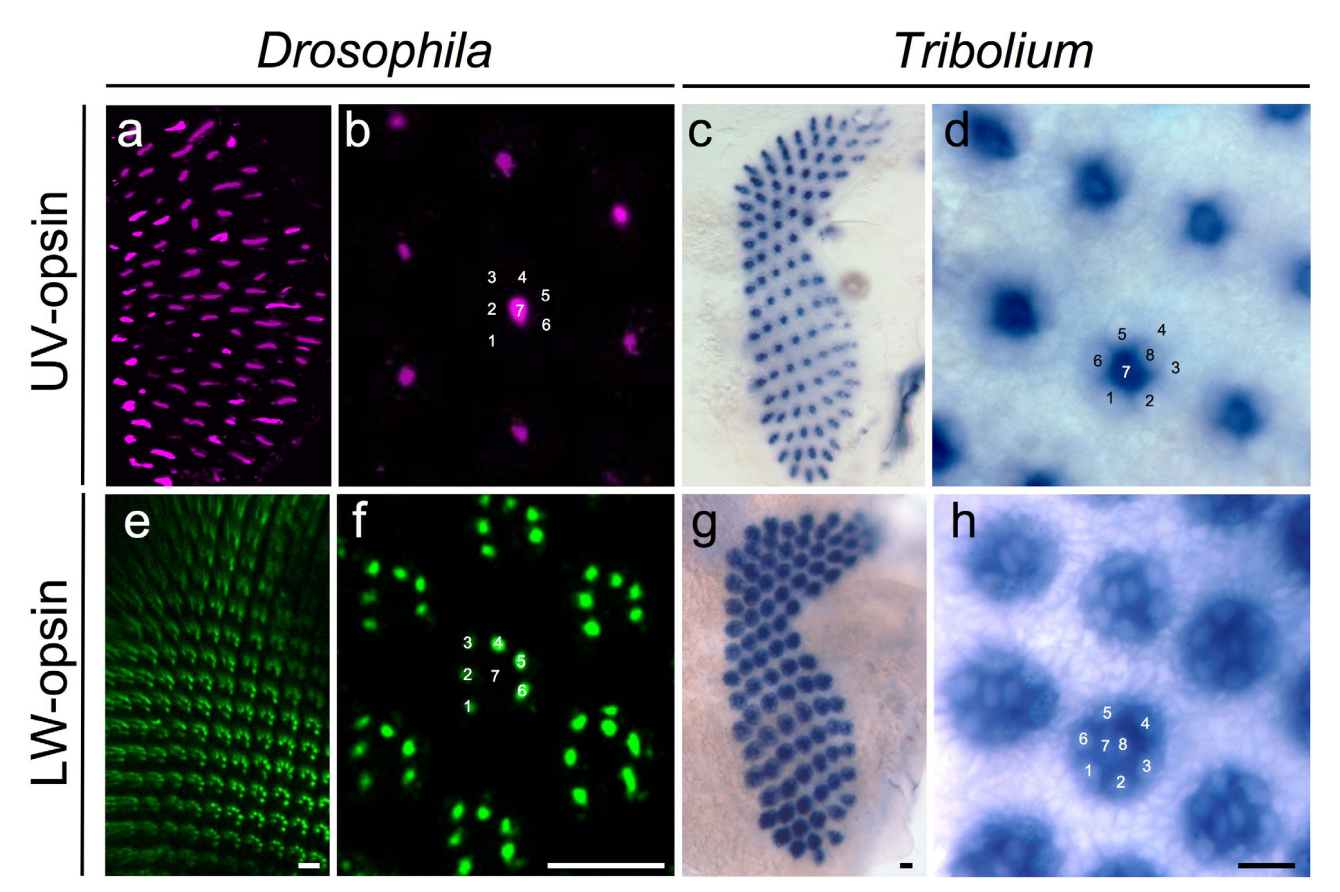
**Differential expression of opsin paralogs in *Drosophila *and *Tribolium***. (a and b) Digital sections of *Drosophila *pupal tissue stained with antibodies against UV-sensitive Rh3 and Rh4 opsins. (a) Low magnification overview. (b) High magnification view. Numbers indicate photoreceptor cell subtypes R1-8. (c and d) *Tribolium *UV-opsin expression detected by *in situ *hybridization. (c) Low magnification overview of UV-opsin expression throughout entire pupal retina. (d) High magnification view of cell specific expression of *Tribolium *UV-opsin. Numbers indicate photoreceptor cell subtypes. (e and f) Digital sections of *Drosophila *pupal tissue stained with antibody against the long wavelength-specific opsin Rh1. (e) Low magnification overview. (f) High magnification view. Numbers indicate photoreceptor cell subtypes R1-8. (g and h) *Tribolium *LW-opsin expression detected by *in situ *hybridization. (c) Overview of LW-opsin expression throughout entire pupal retina. (d) High magnification of cell specific expression of *Tribolium *LW-opsin. Numbers indicate photoreceptor cell subtypes. Scale bars correspond to 10 μm.

Each *Tribolium *ommatidium contains eight photoreceptors, each of which is unambiguously related to one of the photoreceptor subtypes R1–R8 of *Drosophila *by position, rhabdomere structure and ontogenetic origin [13]. We therefore compared the spatial distribution of *Drosophila *UV- and LW-opsins detected by immunohistochemistry with *Tribolium *UV- and LW-opsin as detected by *in situ *hybridization (Fig. 2 and data not shown).

### Spatial regulation of the expression of the *Tribolium *UV opsin gene

We first investigated the expression of *Tribolium *UV-opsin. To generate a *Tribolium *UV-opsin RNA probe for whole mount *in situ *hybridization, we cloned a 502 bp fragment of the predicted *Tribolium *UV-opsin gene. In *Drosophila*, the UV-opsin paralogs Rh3 and Rh4 are stochastically distributed in non-overlapping patterns in all central R7 photoreceptor cells (Fig. 1, Fig 2a and 2b) [14,15]. Similarly, *in situ *hybridization experiments with an RNA antisense probe against *Tribolium *UV-opsin in mature pupal and early adult retina detected strong expression in a single photoreceptor cell per ommatidia throughout the entire retinal field (Fig. 2d). Differential interference contrast microscopy at high magnification confirmed that the *Tribolium *UV-opsin paralog was selectively expressed in all R7-type cells (Fig. 2e). The comparison with *Drosophila *thus revealed that *Tribolium *UV-opsin is expressed in a conserved R7-specific manner.

### Spatial regulation of the expression of *Tribolium *LW-opsin gene

Next we investigated the expression of *Tribolium *LW-opsin. In *Drosophila*, the peripheral photoreceptors R1-6 express the LW-opsin paralog Rh1 (Fig. 2e and 2f), and 70% of central R8 cells express the LW-opsin Rh6 [16]. The remaining 30% of R8 cells express the *Drosophila *B-opsin paralog Rh5 [17,18]. For whole mount *in situ *hybridization in *Tribolium*, we cloned a 972 bp long RT-PCR fragment that includes the entire predicted *Tribolium *LW-opsin ORF from head specific *Tribolium *total RNA. Analysis of the expression of the single *Tribolium *LW-opsin gene revealed uniform transcription in all photoreceptors of the retina (Fig. 2g). Importantly, expression was not only detected in the peripheral photoreceptors, but also in all central R7 and R8 photoreceptors (Fig. 2h). This finding reveals a strongly expanded expression domain of LW-opsin compared to *Drosophila*, and indicates that R7 cells in *Tribolium *co-express the LW and UV-opsin-encoding genes.

Like in most insects, ommatidia in the dorsal rim area (DRA) of the *Drosophila *compound eye exhibit modifications for the detection of polarized light including expression of UV-opsin in both R7 and R8 [19,20]. However, no evidence of regional heterogeneity of opsin expression could be detected in the dorsal region of the *Tribolium *retina (Fig. 2c and 2g), consistent with the lack of morphological evidence of DR ommatidia in this species [13].

## Discussion

Taken together, the new expression data described here reveal a number of unique aspects of the *Tribolium *compound eye retina: (1) lack of B-opsin encoding photoreceptors, (2) homogeneous expression of a single LW-opsin across the entire retina, and (3) co-expression of UV- and LW-opsin in all R7 cells. These findings generate questions regarding the evolutionary mechanisms, forces and pathways that gave rise to the exceptional *Tribolium *retinal mosaic.

### *Tribolium *LW- and UV-opsin expression is consistent with loss of B-opsin

**Figure 3:**
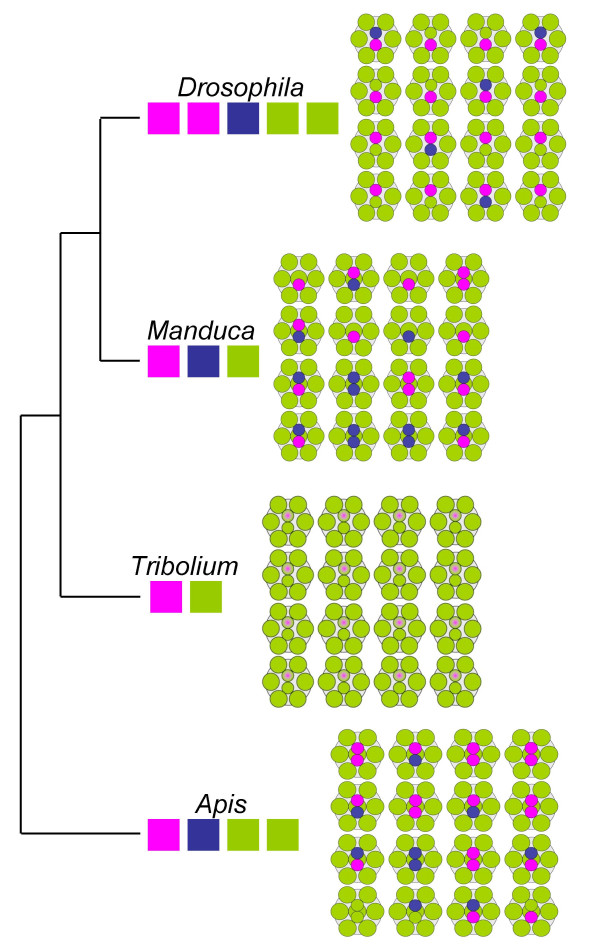
**Comparison of insect photoreceptor mosaics**. Schematic presentation of photoreceptor sensitivity arrays in the compound eyes of honeybee (*Apis*), tobacco hornworm moth (*Manduca*), *Drosophila*, and *Tribolium*. Colored boxes underneath genus names indicate number and wavelength specificities of opsins that are known to be expressed in the main retina. Tree visualizes phylogenetic relationships between the insect orders to which the four species belong [51]. In the retinal mosaics, colors indicate photoreceptor light specificities in either the UV (violet), blue or green wavelength range. *Drosophila *forms eight photoreceptors per ommatidium. The peripheral photoreceptors R1-6 express a LW-opsin. 70% of the Drosophila ommatidia are of the yellow type, in which the central R8 cell express a the LW-opsin paralog Rh6 and the central R7 cells the UV-opsin Rh4. In the remaining pale-type ommatidia, the central R8 cell express the B-opsin Rh5 and the central R7 cells the UV-opsin Rh3 (Fig. 1) [12]. Honeybee and tobacco hornworm moth develop nine photoreceptors per ommatium due to duplication of the R7 cell fate [52]. However, in both species only the two central R7-like more distally located cells exhibit differential opsin expression ranging from UV to LW sensitive opsins. The peripheral photoreceptor cell homologs R1-6 express LW-opsin as does the proximally located central R8 cell homolog. Five different ommatidia types can be distinguished in the tobacco hornworm moth retina, which differ by number of R7-like cells (1–2) or the combination of B- and UV-opsin expressing R-7 cells [12]. In the honeybee, six different ommatidia types occur which either express G-, UV- or B-opsin in both R7-like cells or in any possible combination [24]. In Tribolium, LW-opsin is expressed in all photoreceptor cells. Co-expression of LW- with UV-opsin in R7 is indicated by gradient from violet to green.

The expression of the *Tribolium *LW- and UV-opsins accounts for all photoreceptors in the *Tribolium *retina, and is compatible with a loss of B-opsin in the flour beetle. This finding further cements evidence for color vision reduction in the evolutionary lineage leading to *Tribolium *(Fig. 3). Consistent with this, the spectral sensitivity of the very closely related mealworm *Tenebrio molitor *has been reported to peak only in the UV and green wavelength ranges based on electroretinogram measurements [21]. Moreover, preliminary PCR screens from *Tenebrio molitor *genomic DNA resulted in isolation of closely related orthologs of *Tribolium castaneum *LW- and UV-opsins, while similar attempts to isolate B-opsin orthologs failed, just as in *Tribolium castaneum* (Bao and Friedrich, unpublished). Hence genome, gene expression and physiological data converge on supporting the loss of B-opsin in *Tribolium castaneum*.

### Loss of B-opsin expressing photoreceptors through expansion of the LW-opsin expression domain: *cis*-regulatory implications and possible trajectories

Our data reveal that the lack of B-opsin in *Tribolium *correlates with a dramatic expansion of LW-opsin expression. In most endopterygote retinas studied so far, LW-opsins are expressed in the peripheral photoreceptors R1-6 and a fraction of central R7 or R8 cells (Fig. 3). Thus, the expanded LW-opsin we observe in *Tribolium *is likely to have played an essential role in the evolution of its retinal mosaic. The comparative data further imply that these changes involved the expansion of the peripheral LW-opsin expression domain into R7 and R8.

A most accurate inference of the underlying mutational events underlying the *Tribolium *opsin expression pattern depends on knowing the organization of the retinal mosaic in the last B-opsin expressing ancestor of *Tribolium*. Although this is still much a matter of speculation, some conclusions can be drawn from physiological data from other Coleoptera and opsin expression data in other endopterygote orders. Based on electrophysiological studies, for instance, species in the beetle families Carabidae (ground beetles) and Coccinelidae (lady beetles) are equipped with sensitivities in the green, blue and UV [22,23]. Considering further that B-, LW- and UV-opsins are expressed in R7 and R8 cells in Hymenoptera, Lepidoptera and Diptera, it is most likely that *Tribolium *evolved from an ancestor in which R8 and R7 cells differentially expressed combinations of at least three opsins, similar to *Drosophila *(Fig. 3) [5,11,24].

**Figure 4:**
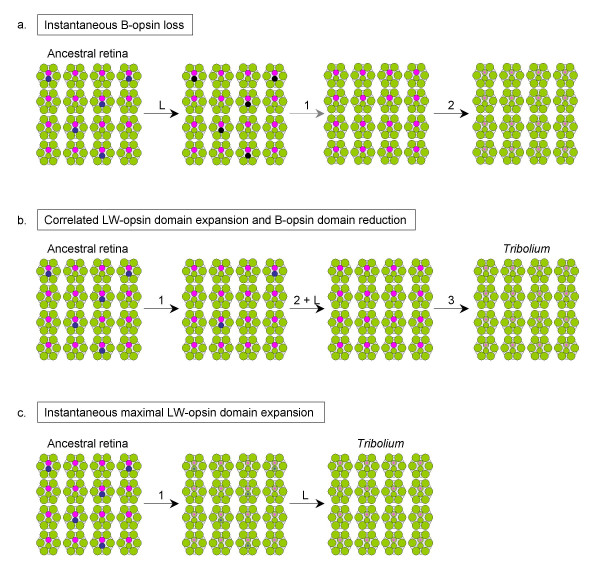
**Possible trajectories of *Tribolium *retina evolution**. Schematic presentation of three mutation scenarios that could have led from an ancestral trichromatic retina to the retinal mosaic of extant *Tribolium castaneum*. Colors indicate photoreceptor light specificities as in Fig. 3. Numbers above arrows represent number of necessary mutation induced gene regulatory changes underlying opsin expression pattern evolution. L stands for genomic loss of B-opsin. (a) Instantaneous B-opsin loss scenario. B-opsin loss without correlated or preceding expansion of other opsin expression domains is unlikely since it would result in a fraction of light insensitive photoreceptor cells and thereby a substantial deterioration of the resolution of spatial vision (black). Subsequent mutational steps that would lead to the *Tribolium castaneum *retinal mosaic are therefore just indicated with increased opacity. Genomic B-opsin loss was more likely preceded by either gradual expansion of the LW-opsin expression domain (b) or immediate maximum expansion of LW-opsin expression (c). See text for further details.

Based on this premise, it is reasonable to assume that the UV-opsin expression in R7 is a conserved aspect of the *Tribolium *retina, whereas the loss of B-opsin expression and correlated expansion of LW-opsin expression is derived. This latter change may have occurred through one of several possible trajectories (Fig. 4). First, the loss of B-opsin expression could have been initiated through mutations that abrogate transcriptional activation or expression of a functional protein prior to or independent of changes in the ancestral expression domain of LW-opsin (Fig. 4a). However, this would create a retinal mosaic with partially light-insensitive photoreceptor cells. Considering the importance of flight in *Tribolium *(see below), the ensuing deterioration of spatial vision would likely be a devastating fitness penalty. This suggests that the molecular evolution of B-opsin loss was correlated with, or contingent upon, compensatory changes in the regulation of LW-opsin, a second possibility (Fig. 4b).

Several mechanisms exist regarding the nature of such *cis*-regulatory changes in the B- and LW-opsin genes. Assuming heterogeneity of LW-opsin and B-opsin expression among ancestral R8 cells, for instance, correlated mutations in the *cis*-regulatory regions of both genes may have led to a gradual increase in the fraction of LW-opsin expressing cells that eventually culminated in the complete replacement of B-opsin expressing cells. This scenario describes a model of gradual replacement of blue versus green-sensitive photoreceptors in which color vision evolves as a quantitative trait (Fig. 4b). Alternatively, *cis*-regulatory change may have resulted in immediate compensatory loss of B-opsin and expansion of LW-opsin expression. This type of change could have been induced by separate mutations in the *cis*-regulatory regions of both genes or, as data from *Drosophila *suggest (see below), through mutational change in the *cis*-regulation of only one opsin pending pre-existing mechanisms of mutual repression. Both trajectories require at least one additional regulatory change that would allow further expansion of LW-opsin expression into R7 (Fig. 4b).

A third scenario is that a single mutation led to the immediate expansion of the LW-opsin expression into all photoreceptor cells. The consequence would be that of co-expression of LW-opsin with B-opsin in a fraction of R8 cells, and with UV-opsin in R7 cells (Fig. 4c). Such a retinal mosaic would thus be one step away from that in extant *Tribolium *by mutational disintegration of the activating elements in the ancestral B-opsin *cis*-regulatory region. This pathway seems the most probable for three reasons: (1) requiring only a single mutation to generate the final LW-opsin pattern is most parsimonious, (2) the co-expression of LW- and UV opsins in the R7 cells of *Tribolium *is consistent with this scenario, and (3) the latter fact as well as data from other species (see below) demonstrates that opsin co-expressing photoreceptors are viable and functional. Moreover, a strikingly similar scenario exists in *Drosophila*, where a single point mutation in the LW-opsin promoter, Rh6, or in the transcription factor, *Orthodenticle *(*Otd*) leads to expansion of Rh6 (and co-expression of opsins) into the majority of peripheral photoreceptors and many R7 central photoreceptors, but not into the B-opsin-containing R8 cells [25].

### Possible mechanisms for LW-opsin expansion: lessons from *Drosophila*

Molecular genetic analyses in *Drosophila *have unraveled several critical mechanisms of *cis*-regulatory control of opsin expression, some of which are likely conserved [5,12]. Those particularly relevant to the discussion of the evolution of the *Tribolium *retina involve studies on the regulation of the LW-opsin-encoding gene, *Rh6*. Such studies have demonstrated that at least four independent transcriptional repression processes normally restrict *Drosophila *Rh6 to 70% central R8 photoreceptors. In R8 cells, the mutually exclusive B- versus LW-opsin expression in 30 vs 70% R8 cells occurs through a bistable loop of both cell signaling and transcriptional regulation [26]. Failure to properly initiate this loop leads to a retina that expresses all B-opsin or all LW-opsin genes in R8 cells. Interestingly, LW-opsin expression is the "default" R8 opsin state, whereas B-opsin requires induction. Thus, this regulatory mechanism could reflect that the loss of B-opsin observed in *Tribolium *is secondary to a loss in this induction process. This regulatory mechanism also suggests an inherent capacity of the photoreceptor mosaic in higher flies to evolve along the gradual replacement model considered for *Tribolium *above. Second, *Drosophila *express two LW-opsins: *Rh1 *in peripheral non-color sensitive photoreceptors, R1-R6, and *Rh6 *in 70% R8 cells. Co-expression of both LW-opsins is specifically prevented in peripheral photoreceptors due to cell-specific repression of *Rh6 *[25]. A third mechanism of repression for *Rh6 *occurs in all R7 cells, where it is repressed by the R7-specific transcription factor, Prospero (Pros) [27]. Finally, *Rh6 *expression in DRA ommatidia (both R7 and R8 cells) is prevented through the selective inactivation of the R8-specific, *Rh6 *activating transcription factor Senseless (Sens) in the DRA [28]. Thus, the release of analogous LW-opsin repression mechanisms could have led to the expansion of LW-opsin expression as postulated for the evolution of *Tribolium*.

While it is premature to draw final conclusions on the trajectory of *cis*-regulatory changes that generated the retinal mosaic of *Tribolium castaneum*, data and implications identify *Tribolium *as unique model to study compensatory gene regulatory mechanisms associated with gene loss. Progress in this area can be made by studying the *cis*-regulatory control of *Tribolium *LW-opsin in comparison to that of *Drosophila Rh6 *and *Rh1*, and by exploring the conservation of opsin expression in a wider range of darkling beetles, as well as in distantly related Coleoptera.

### Consequences and significance of opsin co-expression in the *Tribolium *R7 cells

Across orders, the "one receptor rule" is important for sensory receptor expression [3,29]. The only two exceptions to this rule in insect photoreceptors reported thus far occur in butterfly species that have regionalized photoreceptors that co-express two opsins [30,31]. Thus, *Tribolium *is the first insect example in which a specific photoreceptor subtype co-expresses two different opsins across the entire retina. Opsin co-expression is more common in vertebrates than invertebrates [32]. In the Siberian hamster and the pouched mouse, for instance, dual opsin-expressing cone cells are uniformly spread throughout the retina similar to *Tribolium *[33]. Intriguingly, vertebrate species with exceptionally high frequencies of opsin co-expressing photoreceptors are either nocturnal or fossorial [32,34]. This evolutionary parallel raises the possibility that opsin co-expression is of advantage under conditions where brightness sensitivity is critical. This bodes well with the low light ecological niche of *Tribolium *since all postembryonic stages of *Tribolium *show strong negative phototaxis [35,36].

Both vertebrate and insect opsin co-expressing photoreceptors have been found sensitive to a light spectrum that equals the summation of the single sensitivities of the expressed opsins [30,37,38]. While the excitation characteristics of the *Tribolium *R7 photoreceptors await to be analyzed by electrophysiological means, it is reasonable to assume that these cells are sensitive to a broad range of light from the green into UV wavelengths. What then may have caused the evolution of this unusual trait? For this question to be answered, it is necessary to further explore the functional consequences of opsin co-expression and, secondly, the state from which the *Tribolium *patterns originated.

Modeling data suggest that optimal single opsin-containing photoreceptors exhibit higher photon capture rate than dual opsin-expressing photoreceptors [6]. However, physiological measurements and ecological correlations point to a stronger impact of extended wavelength sensitivity for enhancing brightness detection. In house fly, for instance, the LW-opsin-expressing peripheral photoreceptors R1-6 contain a UV sensitizing pigment [39], which leads to an expanded wavelength sensitivity similar to that assumed for the *Tribolium *R7 cells. Importantly, this supplemental UV-sensitizing pigment has been shown to significantly increase overall brightness sensitivity in the peripheral photoreceptors of the house fly. The effect is considered to be particularly significant under low light conditions [39]. Interestingly, *Tribolium castaneum *does disperse by flight under conditions of stress or exposure to strong light [40]. The conservation of flight capability indicates that flight has maintained fitness importance throughout *Tribolium *evolution. Moreover, adults of the meal worm *Tenebrio molitor *are negatively phototactic like *Tribolium *but become positively phototactic after extended times of desiccation [41], suggesting that this closely related species to *Tribolium *behaves as a facultative cryptozoic species. Thus, the *Tribolium *visual system may have been molded by long-term cryptozoic adaptation.

From which functional ancestral state did the broadband sensitivity of *Tribolium *R7 cells evolve? While the function of the *Tribolium *R7 photoreceptors may have changed to extreme broadband photoreceptors to boost the overall photon capture capacity of the retina, enhanced brightness sensitivity is not incompatible with maintenance of color detection capacity. In theory, excitation differences between LW-opsin expressing R8 and LW/UV-opsin-coexpressing R7 cells may still discriminate UV from green patterns to facilitate dichromatic vision in *Tribolium*. The former may have evolved without completely sacrificing UV versus LW discrimination ability. Indeed, structural and comparative evidence suggest that the *Tribolium *central photoreceptor cells R7 and R8 represent ancestral color detectors. The cellular architecture of the *Tribolium *ommatidia is very similar to that in mealworm *Tenebrio molitor*. In both species, the six peripheral photoreceptors form a ring of rhabdomeres while the inner R7 and R8 photoreceptors contribute a separate rhabdom stem [13,42]. Importantly, *Tenebrio *ommatidia exercise pupil-like brightness adaptation through volume changes of the primary pigment cells which sit on top of the photoreceptor bundle [42]. In the light-adapted state, the primary pigment cells are expanded such that only the central R7 and R8 cells are exposed to the light path. In the dark-adapted state, the primary pigment cells further contract to additionally expose the peripheral R1-6 to light. This mechanism suggests specialization or division of brightness detection versus color discrimination tasks between inner and outer photoreceptors, similar to *Drosophila*. While this interpretation remains to be experimentally tested, the fact that the *Tenebrio *light adaptation mechanism is conserved in the more distantly related darkling species *Zophobas atratus *strongly suggests that the *Tribolium *situation evolved from a central photoreceptor-based color discriminating system [42].

Taken together, there is circumstantial evidence for both costs and benefits to opsin co-expression in *Tribolium *R7 cells. Thus, it will be necessary to elucidate the relative significance of these aspects for the biology of *Tribolium *to understand the evolutionary forces that generated the *Tribolium *retinal mosaic. Importantly, while dispersal by flight under conditions of stress seems to represent the likeliest condition under which brightness sensitivity may be critical for *Tribolium*, observations in the laboratory suggest little dependence of food and mating partner detection on vision [35]. Nonetheless, the cryptozoic habits of *Tribolium *speak for an increased selection pressure on brightness sensitivity and relaxation of the selection pressure on color discrimination needs.

### Neutral or adaptive replacement of B-opsin?

The most parsimonious trajectory of *cis*-regulatory evolution that leads to the retinal mosaic of extant *Tribolium *is mechanistically linked to both the emergence of UV-/LW-opsin co-expressing R7 cells and the loss of B-opsin expression in R8 cells (Fig. 4c). The potential adaptive advantage of UV-/LW-opsin co-expression in *Tribolium *R7 cells generates the question: could the loss of B-opsin have been influenced not only by relaxation of color vision needs but also enforced by an adaptive effect of increased brightness sensitivity? Lack of blue sensitivity is reliably documented in only a few cases of insects, including the crepuscular cockroach *Periplaneta americana*, and the owlfly *Ascalaphus macarionius*, a diurnal predator [43,44]. The diverse behavioral patterns of these species, however, have provided little information regarding the potential causes leading to reduced color discrimination [2]. Remarkably, in vertebrates, opsin dual-expressing photoreceptors as well as single LW-sensitive photoreceptors are increased in species with cryptozoic life histories (for review see [34]). Keeping in mind that brightness detection is primarily based on LW-opsins in both vertebrates and insects, the parallel increase of LW-opsin expressing cells in cryptozoic vertebrates and *Tribolium *is further suggestive of an adaptive trend. The replacement of B-opsin with LW-opsin expressing cells during *Tribolium *evolution may thus have been adaptive. However, this does not enforce the conclusion that the loss of *Tribolium *B-opsin involved positive selection. Either gradual or complete expansion of the LW-opsin followed by decay of B-opsin expression may have occurred as neutral or permissively deleterious changes in color vision lost fitness importance, while brightness sensitivity remained under purifying selection in the lineage leading to *Tribolium*. It is not clear whether the similarity in the opsin repertoires of cryptozoic insect or vertebrate retinas reflects convergent preferential conservation of photoreceptors essential for brightness detection or adaptive enrichment within a short time window of cryptozoic adaptation [34]. Extended comparative data may provide further insights. For instance, conservation of specific UV-opsin expression but not LW- and UV-opsin co-expression in R7 cells in related darkling beetles would indicate a persisting need of detecting UV light patterns but point against a strong advantage of brightness sensitivity increase through LW- and UV co-expression. Conserved co-expression of UV with LW-opsin in R7, on the other hand, would be most reasonably correlated with a fitness advantage of maximizing brightness sensitivity.

## Conclusion

With many questions awaiting to be addressed, it seems clear that the first look into the retinal mosaic of a cryptozoic coleopteran marks just the entry point for studying the dark side of insect vision. Besides serving as a model for development and pest biology, which provided the rationale for genome sequencing [45], *Tribolium *also holds promise to add illuminating data to the classic debate over the mechanisms of reductive phenotype evolution in cryptozoic animals [46].

## Methods

### Animal stocks

*Pearl *pBac(3xP3-EGFP)af *T. castaneum *were reared on 5% yeast supplemented whole wheat flour at 31°C in constant darkness. *y*^1^*w*^67^*Drosophila melanogaster *lines were maintained on standard cornmeal-molasses food at room temperature.

### Bioinformatics

The putative coding regions of *Tribolium castaneum *LW-opsin and UV-opsin were identified by TBLASTX search with the *Drosophila *Rh1 and Rh3 gene sequences to the *Tribolium castaneum *Georgia GA2 genome assembly (release 8/17/2005), accessed through the Human Genome Sequencing Center server in the Department of Genetics at Baylor University [47]. Similar searches with the remaining *Drosophila *opsins, Rh2, Rh4-Rh6, failed to identify additional *Tribolium *opsin-encoding genes.

### Molecular biology

A 502 bp fragment of the predicted *Tribolium *UV-opsin gene was amplified from *Tribolium *total genomic DNA by standard PCR using the specific primers 5'-CCCCAAAGACGAACTCATTC-3' and 5'-ATGGTGTACCCCCAAACAAA-3'. This primer combination produces a PCR amplicon that extends from the first putative open reading frame (ORF) containing exon of the *Tribolium *UV-opsin gene to the second ORF-containing exon bridging a 55 bp intron. 972 bp of the predicted *Tribolium *LW-opsin ORF were amplified by nested RT-PCR using outer primer pair TcRhR-3F (5'-TCACCGTCGTGGACAAAGTC-3') and TcRhR-3R (5'-TTCTCGTCGGTGGTAACGTTC-3'), and inner primer pair TcRhR-4F (5'-GCCCATTGGTACCAGTTCC-3') and TcRhR-4R (5'-AGCCGTGGAGGTGGTGTC-3'). Total RNA was extracted from adult *Tribolium *head with the RNAqueous kit (Ambion). The RETROscript kit (Ambion) was used to generate cDNA through reverse transcription initiated with random decamers. The resulting PCR fragments were cloned into pGEM^®^-T Vector (Promega). Multiple identical sequences were obtained for each paralog. The sequences are accessible under accession numbers [GenBank: DQ029113] and [GenBank: EU164547].

### *In situ *hybridization, immunohistochemistry, and microscopy

95% pupal *Drosophila *whole-mounted retinas were dissected at room temperature (RT) in PBT (1 × PBS + 0.1% Triton X100, pH 7.2), fixed in PLP (1 × PBS, 4% paraformaldehyde, 0.075 M lysine, 0.01 M sodium periodate, 0.05% saponin) [48], and washed 3 × 10 min with PBT. Retinas were transferred to Signal-iT FX (Invitrogen) for 30 min, RT, before incubation with mouse anti-Rh1 (1:100, DHSB), mouse anti-Rh3 (1:5, gift from S.G. Britt), and rabbit anti-Rh4 (1:250, gift from C.S. Zuker) overnight at 4°C in BNTS (1 × PBT, 1.5 M NaCl, 0.1% BSA, 0.05% saponin). Samples were washed 3 × 20 min with PBT, incubated for 2 hrs at RT with AlexaFluor-488, -555, or -648-conjugated secondary antibodies (1:500, Molecular Probes), and washed thrice again with PBT. Retinas were mounted in Prolong Gold antifade-reagent (Invitrogen), and imaged 24 hr later using an Axioplan IIie equipped with the Zeiss ApoTome deconvolution module. Digital images were processed with Axiovision 4.5 (Zeiss) and AdobePhotoshop 7.0 software.

Whole mount *in situ *hybridization of *Tribolium *pupal retinae was carried out following previously published protocols with minor modifications [49,50]. Digoxygenin-labeled antisense RNA probes were prepared with DIG-UTP RNA Labeling Mix (Roche). Pupal retinas were dissected in ice cold PBS, and fixed in 9.25% formaldehyde, 50 mM EGTA ph8, and 10% Triton X in PBT at RT for 1 h. Tissues were then washed one time in PBS, three times in PBT, and equilibrated for hybridization with a 10 min incubation in 50% PBT+50% Hyb-A-RNA (hybridization buffer A) at RT, and 1 h at 55°C in 100% Hyb-A-RNA (50% formamide, 4 × SSC, 250 μg/ml tRNA, 0.1% Tween-20, 1 × Denhardt's solution, 5% dextran sulfate) with 500 μg/ml freshly denatured herring sperm DNA. Hybridization was carried out for 72 h in Hyb-A-RNA with RNA probe at approximately 0.5 μg/ml concentration at 55°C. Subsequently, pupal retinas were washed 3 × 30 min in Hyb-B-RNA (50% formamide, 4 × SSC, 0.1% Tween-20) at 55°C followed by a 24 hrs final washing step. Next, tissues were equilibrated in 5 × SSC at RT for 5 min and another 5 min in maleic acid buffer (0.1 M maleic acid, 0.15 M NaCl, 0.1% Tween-20). Tissue was blocked for 2 hrs at RT in maleic acid buffer supplemented with Roche *in situ *blocking buffer at 1:10 dilution (Roche). Anti-DIG antibody (Roche) was added at a 1:5000 dilution and tissues were incubated 2 hrs at RT on a shaker. Following antibody incubation, pupal eye discs were washed 3 × 1 hrs. Tissues were then transferred into detection buffer (0.1 M Tris-HCl pH 9.5, 0.1 M NaCl, 50 mM MgCl_2_) for 5 min at RT. Color detection was initiated by adding 4.5 μl NBT (50 mg/ml) and 3.5 μl BCIP (50 mg/ml) as substrate. Color reactions were developed for 1 to 2 hrs and stopped by replacing staining buffer with PBT. Labeled retinas were washed with EtOH, rehydrated, and cleared in 70% glycerol in PBS before mounting. DIC brightfield images were recorded through a Zeiss Axioscop coupled to a SPORT RT camera (Diagnostic Instruments). Contrast and brightness were adjusted in Photoshop CS3.

## Competing interests

The author(s) declare that they have no competing interests.

## Authors' contributions

MJ carried out *Tribolium *LW- and UV-opsin expression analysis, RB cloned the *Tribolium *UV-opsin gene fragment and carried out gene ortholog searches, ZL cloned the *Tribolium *LW-opsin RT-PCR fragment, ECM generated *Drosophila *opsin expression data, TAC designed analysis of *Drosophila *opsin expression and co-wrote the manuscript, MF conceived the study and wrote the manuscript.
